# Emergency dispatch, FirstAED global positioning of first responders with distinct roles - a solution to reduce the response times and ensuring early defibrillation in the rural area Langeland

**DOI:** 10.1186/1757-7241-23-S1-A4

**Published:** 2015-07-16

**Authors:** Henrik Schakow, Mogens L Larsen, Finn L Henriksen

**Affiliations:** 1 The AED Center, Department of Cardiology, OUH Odense University Hospital, Odense, Denmark; 2 Department of Health Science and Technology, Aalborg University, Aalborg, Denmark

## Purpose

FirstAED is meant as a supplement to the existing emergency response systems. The purpose is to shorten the first responder response times at emergency calls to below 5 minutes on the island of Langeland. The FirstAED project defines a new way to dispatch the nearby first responders and organize their roles in the hope of reducing response times and improving survival rates.

## Methods

First aid and cardiopulmonary resuscitation is provided by 215 first responders who use their smartphone (iPhone 4S/5). The population purchased 95 AEDs which are available around the clock and placed less than two kilometres apart. FirstAED is an auxiliary to the public services and it enables the emergency dispatcher to send an organized team of first responders with distinct roles to the scene. FirstAED global positioning system GPS-track the 9 nearby first responders. FirstAED chooses the 3 most optimally located first responders who have accepted the alarm. FirstAED organizes the three first responders in a team: no. 1 reaches the patient to give cardiopulmonary resuscitation; no. 2 brings the AED; and no. 3 is the onsite coordinator.

## Results

During the first 21 months the FirstAED GPS system was used 588 times. Three first responders arrived in 89% of the cases, and they arrived before the ambulance in 95% of the cases. FirstAED entailed a significant reduction in median response time from more than 8 minutes before to 4 minutes and 9 seconds after. The first responder was on site in less than 5 minutes in more than 60% of the cases. The AED was on site within a median time of 5 minutes and 58 seconds. The first responders were on behalf of their short response time involved in many different cases: cardiac arrest, disease, accidents, traffic accidents, fire, subarachnoid haemorrhage, hanging, divers' decompression sickness, and sea rescue.

## Conclusions

GPS-tracking reduces the response times, and the quality of the effort improves as all the first responders who accept the FirstAED alarm have distinct roles.

